# Surface microbial dynamics and residual diversity in patient rooms with wooden and painted walls

**DOI:** 10.1038/s41598-026-64982-y

**Published:** 2026-08-07

**Authors:** Anastasiia Postovoitova, Kateryna Davydenko, Larysa Kucher, Olov Karlsson, Olena Myronycheva

**Affiliations:** 1 Division of Wood Science and Engineering, Luleå University of Technology, 93187 Skellefteå, Sweden; 2 Department of Population Genetics, Institute of Food Biotechnology and Genomics, National Academy of Sciences of Ukraine, Kyiv, 04123 Ukraine; 3 Department of Forest Mycology and Plant Pathology, Swedish University of Agricultural Sciences, 75007 Uppsala, Sweden; 4 Department of Agrobiology, National University of Life and Environmental Sciences of Ukraine, Kyiv, 03041 Ukraine

**Keywords:** Hospital hygiene, Wall material, Microbial dynamics, ATP measurement, Microbial biodiversity, Ecology, Ecology, Microbiology

## Abstract

Wood is a biobased material that supports long-term carbon storage; however, its suitability for use in healthcare facility interiors remains insufficiently studied. This study assessed surface microbial dynamics and residual diversity in patient rooms with conventional painted wall materials and with designer wooden wall panels. Surface microbial dynamics were evaluated using adenosine triphosphate (ATP) measurements and the swab method with colony-forming unit (CFU) counting. Bacterial and fungal cultures from each patient room were analysed by Sanger sequencing. No significant differences in ATP levels or microbial quantities were observed between rooms. In addition, no correlation was observed between ATP levels and CFU counts within each patient room due to methodological differences. In total, 37 microbial species (24 bacterial and 13 fungal) from 23 genera, all belonging to risk groups 1 and 2, were identified. Significant differences in microbial biodiversity were observed among the patient rooms studied. Fewer unique taxa were detected in the room with wooden walls. This study suggests that the installation of wooden wall panels was not associated with increased surface microbial contamination or altered microbial dynamics under the conditions investigated. The findings support the careful consideration of wood as a finishing material in hospital facilities, while highlighting the need for further replicated studies to confirm these observations.

## Introduction

Hospitals are complex public health institutions that must meet numerous standards and requirements to provide high-quality treatment, surgery, and patient care. In addition to high-quality medical services, good interior design of hospital facilities is also a factor that positively affects the well-being of patients, their family members, and medical staff. Improving the visual appearance of healthcare settings and rooms through new materials, surface finishes, interior elements, and artwork enhances well-being and mental health outcomes, provides distraction, and evokes positive emotions^1–3^. Together, these factors contribute to a more positive hospital experience.

Today, a pressing issue is the selection of building and interior materials based on their environmental impact^4^. The use of biobased materials in the construction industry provides carbon sequestration and long-term storage and makes a significant contribution to the modern global bioeconomy^5^. Wood, as an organic and renewable material, has significant advantages over plastic, glass, concrete, and steel, which require substantial energy for production. These advantages include wood’s favourable environmental profile and its absence of environmental disturbances, pollution, or hazards to human health^6^. It has also been found that using wood in buildings reduces embodied energy by 43% and carbon emissions by 68% compared to using concrete^4^. Despite these benefits and widespread use of wood in furniture and panel manufacturing, its use in healthcare facility interiors remains limited due to concerns related to hygiene, durability, and safety.

Hospitals, like any indoor environment, host complex microbiomes influenced by many factors, including construction materials and interior design^7^. Wood is a hygroscopic, porous material, and its use in high-humidity environments can lead to the accumulation and spread of unwanted microflora. Pathogenic bacteria and fungi present in hospital microbiomes can interact with patients and healthcare staff and may contribute to healthcare-associated infections in susceptible patients^8^. Although infection transmission often occurs from person to person, contaminated surfaces can also serve as a transmission pathway. Consequently, the hygienic characteristics of the interior materials used in hospitals are critically important and of heightened public interest in light of the recent COVID-19 pandemic. The introduction of new materials or changes to existing materials in a hospital interior can negatively affect the environment and its microbiome, potentially causing adverse consequences for patients^9^. That is why the potential introduction of wood as a material for hospital facilities prompts reflection on effective surface cleaning and maintenance, durability, and fire safety^10^.

Today, the most widely used methods for assessing the hygienic status of surfaces in public facilities are the swab method and a method based on measuring the amount of adenosine triphosphate (ATP). The swab method, expressed as Colony-Forming Units (CFU), estimates the number of viable microorganisms (bacteria, fungi, etc.) on the surface and provides a measure of microbial quantities or load^11^. ATP measurement quantifies the total amount of all types of organic contaminants and surface debris, including both microbial and organic contamination sources, such as skin particles, body secretions, and food fragments. ATP level, expressed in relative light units (RLU), provides an instant, relatively inexpensive measure of the surface cleanliness^12,13^. In general, these two methods are often used both independently and in combination and are indispensable for hygienic surface monitoring in hospital facilities^11^.

Considering all of the above, the use of designer wooden wall panels in hospital patient rooms represents an emerging interior design solution with potential environmental and well-being benefits. However, the potential impact of new wood materials on the hygienic status and microbiome of these environments had not been investigated. We hypothesised that the presence of wooden wall panels in hospital patient rooms influences the composition and dynamics of surface microbial communities, leading to measurable differences in microbial diversity compared with an otherwise identical room without them. Therefore, the main aim of this work was to assess surface-year-round microbial dynamics and the resulting residual diversity in two identical patient rooms at Skellefteå Hospital (Sweden) that differed in the presence of wooden wall panels in one room.

## Materials and methods

### Characteristics of patient rooms

Two patient rooms in the orthopedic ward at Skellefteå hospital (Skellefteå, Sweden) were selected for this study. The rooms had identical floor area (around 129 m^2^), orientation (south-facing windows), and number of windows (two per room). The supply and exhaust airflow were 100 l/s and 52 l/s, respectively, in both rooms. The patient rooms were Class 2 hygiene premises, specifically designed for all patient care, treatment, and reception activities. According to hygiene class 2 requirements, all interior surfaces in the room must be washable and wipeable^14^. Given this, cleaning in both rooms was carried out according to a daily schedule from Monday to Friday, except on Saturday and Sunday, and included wet wiping of surfaces and wet and dry mopping of the floor. To minimise environmental variability between wards, both patient wards performed the same clinical function, had the same floor area, orientation, ventilation rate, hygiene classification, and cleaning schedule and protocol throughout the study period. However, patient numbers, patient turnover, visitor frequency, and daily clinical activities were not controlled for in the study design, as observations were conducted under normal hospital conditions.

**Fig. 1:**
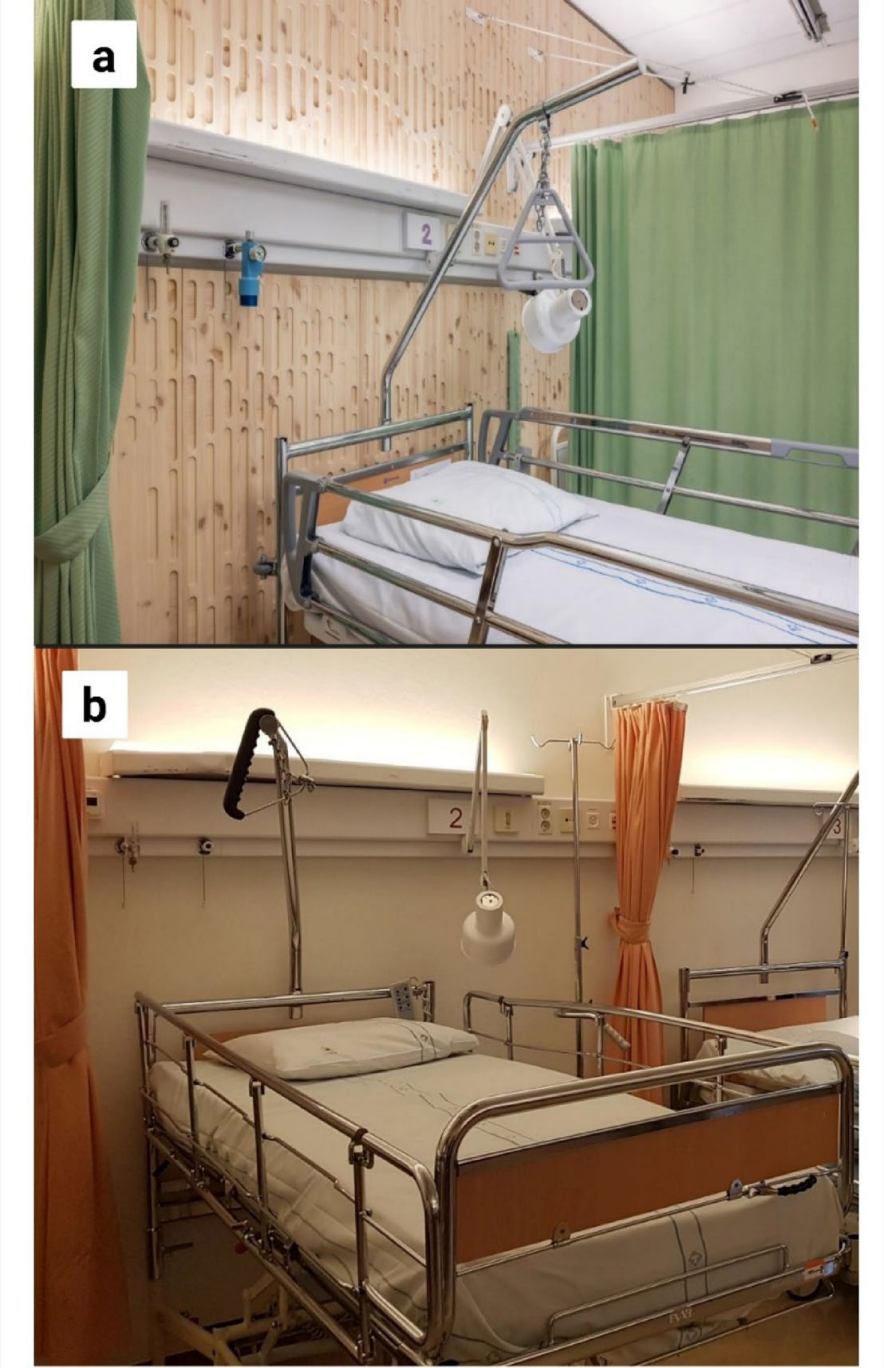
View of the patient rooms in which the study was conducted. (**a**) Room with a wood-panelled wall with a specific design pattern (WoodW). (**b**) Room with only painted walls (PaintW).

All the walls of patient rooms were covered with plasterboard with painted fiberglass. The main difference was that designer wood panels were installed on one wall in one room (hereinafter referred to as **WoodW**) (Fig. 1a), while the corresponding wall in the other room remained entirely painted (hereinafter referred to as **PaintW**) (Fig. 1b).

The wooden panels were made from three layers of Scots pine (*Pinus sylvestris* L.). The panels were 21 mm thick. The specific design pattern shown in Fig. 1a was produced by sawing out the 7 mm-thick top layers. In total, 0.75 m^3^ of Scots pine wood was installed in the WoodW patient room. Before installation, the panels were coated with a transparent, water-based fire-retardant paint NOVATHERM 1FR (Protega AB, Sweden), followed by a clear coat for sealing Top 1FR (Protega AB, Sweden). The manufacturing of the wood panels took about 15 months, with installation completed in September 2021.

### Sample collection

**Fig. 2:**
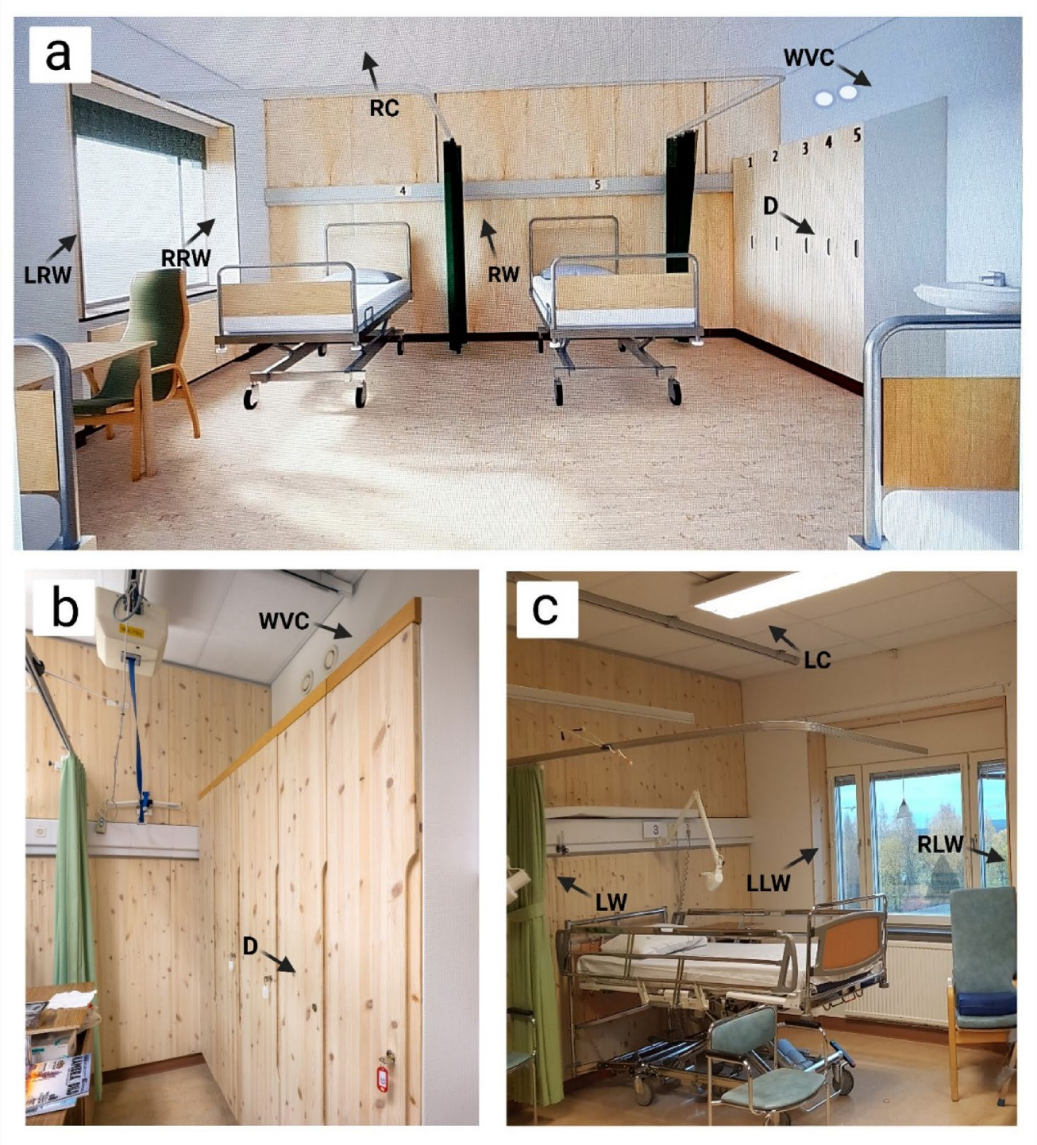
Overview of the hospital patient room with wooden wall panels (WoodW) showing the sampling sites. (**a**, **b**) General view of the right part of the patient room. (**c**) General view of the left part of the patient room. LLW, left (wall) of left window; RLW, right (wall) of left window; LRW, left (wall) of right window; RRW, right (wall) of right window; LW, left wall; RW, right wall; LC, left ceiling; RC, right ceiling; WVC, wall near ventilation channel; D, door of the patient locker. Sample collection in another patient room with painted walls (PaintW) is carried out at the same sites.

Surface cleanliness and microbial contamination were assessed in two patient rooms (WoodW and PaintW) using ATP measurements and surface sampling by sterile cotton swabs. Since the experimental unit in this study was the patient room, multiple sampling locations within each ward and repeated sampling over time were used to characterise spatial and temporal variability within each patient room. Since the wood panels were installed in the WoodW room in September 2021, the sample collection began in October 2021 and continued every 2 months until October 2022. Throughout the entire study period, both patient rooms were in regular use for routine patient accommodation and treatment. A total of 7 measurements were carried out at this stage: October 2021, December 2021, February 2022, April 2022, May 2022, August 2022, and October 2022. At this stage, identification of the microorganisms was not carried out. Ten locations were selected for sample collection in each room (Fig. 2), namely:

- left and right dry walls of the left window (LLW and RLW)
- left and right dry walls of the right window (LRW and RRW)
- left and right walls of the room, about 1.5 m from the floor (LW and RW)
- left and right sides of the ceiling about 1 m from the walls (LC and RC)
- wall near the ventilation channel (WVC)
- door of the patient locker (D)

### ATP measurement

Surface cleanliness was assessed by measuring adenosine triphosphate (ATP) levels directly in both patient rooms using a 3 M Clean-Trace™ Luminometer (Neogen, USA). The sample was collected from the surface (100 mm × 100 mm) using the one-time Clean-Trace Surface ATP Test Swab (Neogen, USA). The wet cotton swab was applied to the surface with repeated horizontal and vertical strokes, rotating the swab tip for 20 s. Following sample collection, the test was activated immediately per the manufacturer’s instructions. The ATP measurement results were displayed in relative light units (RLU). The higher RLU number indicates a more contaminated sample. The ATP benchmark for hospital surfaces was set at 100 RLU, based on previously published data^15,16^.

### Microbial quantification measurement

Microorganisms were collected from surfaces using the swab method according to the Swedish standard SS-ISO 16000-21:2013^17^. A sterile cotton swab was soaked in sterile distilled water and thoroughly wiped the surface (100 mm × 100 mm) for 20 s. The swabbing zones for ATP and microbial quantification measurement were located adjacent to each other but did not overlap. After collecting, the swab was then immediately transferred to a sterile tube and transported to the laboratory. Given the proximity of the hospital and the laboratory, sample transport time was approximately 30 min, and sample processing began immediately upon arrival.

A previously prepared and autoclaved (at 120 °C for 15 min) dilution buffer was added to each tube containing a swab. The buffer consisted of 0.02 M Potassium dihydrogen phosphate (VWR Chemicals, USA), 0.05 M Disodium hydrogen phosphate dehydrate (Merck KGaA, Germany), 0.074 M Sodium chloride (Sigma Aldrich, USA), 0.01% (v/v) Tween 80 (VWR Chemicals, USA), and 1000 ml distilled water. The tubes were shaken for 15 min to wash away cells and spores from the swab. Next, 1 ml of the resulting solution from each tube was transferred to a Petri dish (Ø90 mm) containing sterile malt-extract agar (MEA) (Merck KGaA, Germany) as the cultivation medium, and the solution was evenly distributed over the entire surface of each medium using the same swab. Cultivation was performed in the laboratory chamber (HPP260eco, Memmert, Germany) at 25 °C and 90% relative humidity (RH). The number of colonies on the medium surface was counted daily for 7 days. The results of microbial surface quantities were presented as the total number of Colony-Forming Units (CFU) per sample. If the number of colonies was too high to allow reliable counting by visual inspection, the CFU values were capped at 200.

Based on public recommendations, a threshold of 2.5 CFU/cm^2^ was used to assess the acceptable microbial quantities on hospital surfaces^16,18^. Given the surface area analysed (100 cm^2^) and the washing and inoculation methods for the samples (described above), the CFU count detected on each Petri dish represented the level of microbial contamination per 10 cm^2^ of surface area. For tolerably contaminated hospital surfaces, detected CFU should be no more than 25 CFU per Petri dish (or 2.5 CFU/cm^2^). Accordingly, if 200 CFU were detected on one Petri dish (20 CFU/cm^2^), the surface is highly contaminated with microbial agents.

To identify residual biodiversity of bacteria and fungi species remaining in both studied patient rooms, additional surface sampling was conducted in March 2024, approximately 2.5 years after the installation of the wooden panels in the WoodW room. The protocol for swabbing and washing was identical to that described above. The main difference was that 5 ml of the resulting solution from each tube was inoculated onto five Petri dishes with different cultivation media (1 ml per Petri dish). MEA, potato-dextrose agar (PDA) (Merck KGaA, Germany), nutrient broth agar (NA) (VWR Chemicals, USA), and dicloran 18% glycerol agar (DG 18) (Merck KGaA, Germany) were used as culture media. Subsequently, four Petri dishes containing MEA, PDA, NA, and DG 18 were cultivated in the laboratory chamber (HPP260eco, Memmert, Germany) at 25 °C and 90% RH for 7 days. Additionally, a separate laboratory chamber was used to incubate a Petri dish containing MEA with 1 ml of the resulting inoculated solution at 37 ℃ and 90% RH to detect the growth of thermophilic microorganisms. As negative controls, five Petri dishes containing the appropriate sterile media without the inoculated solution were incubated in parallel with the inoculated dishes under the same conditions.

Following the CFU enumeration, a fragment of colonies with different morphologies was transferred to separate Petri dishes (Ø 45 mm) with the appropriate medium using a sterile needle, and the cultures were further cultivated in the laboratory chamber at 25 ℃ or 37 ℃ and 90% RH for 7 days. A visual inspection for contamination was performed throughout the entire cultivation period, and additional subculturing was carried out when necessary. In this way, both bacterial and fungal pure cultures were isolated and stored at 4 ℃ in a refrigerator until further use.

All culture media were prepared in distilled water according to the manufacturer’s recommendations, then autoclaved (120 °C, 15 min) and poured into sterile plastic Petri dishes (Ø90 mm). All work was carried out in a biosafety cabinet (BSC-700II-I, HMC-Europe, Germany) to ensure aseptic conditions.

### DNA extraction

To identify bacterial and fungal representatives in the WoodW and PaintW patient rooms, total genomic DNA was isolated from pure cultures. DNA extraction was performed using the CTAB protocol^19,20^ with minor modifications. A fragment of mycelium or bacterial colony was transferred to a 2 ml Eppendorf tube with a mixture of silica gel 60H—celite 545 (2:1) (Merck KGaA, Germany), one sterile steel ball (Ø 2.4 mm) and 500 μl of CTAB buffer (200 mM Tris–HCl, 200 mM Na-EDTA, 8.2% NaCl w/v, 2% CTAB w/v, pH 7.5 (Merck KGaA, Germany). The sample was homogenised using a homogeniser (Bead Mill MAX, VWR Chemicals, USA) at maximum speed for 1 min, then incubated at 65 °C for 1.5 h on the block heater (QBD2, Grant, USA). 500 μl chloroform (VWR Chemicals, USA) was added to each tube and mixed vigorously. The samples were then centrifuged in a micro-centrifuge (Micro Star 17R, VWR Chemicals, USA) at maximum speed for 5 min at room temperature, and the supernatant was transferred to clean tubes. The chloroform extraction step was repeated twice. A double volume of cold isopropanol was added, and the solution was left in the freezer (− 20 ℃) overnight for DNA precipitation. The next day, the mixture was centrifuged at maximum speed for 5 min at 4 ℃. The supernatant was discarded, and the formed DNA pellet was washed with 70% cold ethanol, then centrifuged at maximum speed for 5 min at 4 ℃. After removing the supernatant, the pellet was dried in a block heater at 37 ℃ until the ethanol was completely evaporated. The DNA pellet was resuspended in 50 μl TE buffer (10 mM Tris, 10 mM Na-EDTA, pH 8.0 (Merck KGaA, Germany)). DNA concentration was measured using a fluorometer (Qubit Flex, Thermo Fisher, USA) with a Qubit dsDNA BR Assay Kit (Thermo Fisher, USA) according to the manufacturer’s protocol. DNA samples were diluted to 10 ng/μl in TE buffer and stored at 4 ℃ until further analysis.

### Polymerase chain reaction and Sanger sequencing

The polymerase chain reaction (PCR) was performed in a 30 μl reaction mixture using the Thermo Cycler T100 (Bio-Rad, Germany). Each reaction mixture contained 15 μl of DreamTaq PCR Master Mix (2 ×) (Thermo Fisher, USA), 10 pmol of forward and reverse primers (Merck KGaA, Germany), 10 ng genomic DNA, and nuclease-free water (Thermo Fisher, USA).

For bacterial identification, the *16S rRNA* gene was used as the primary target for subsequent sequencing. Primers 27F and 1492R were used to amplify the *16S rRNA* gene fragment containing the hypervariable regions (V1–V9)^21,22^. The PCR protocol consisted of an initial step for 5 min at 94 ℃, followed by 35 cycles of a denaturation step for 30 s at 94 ℃, a primer annealing step for 30 s at 55 ℃, and an elongation step for 1 min at 72 ℃. The final elongation was 7 min at 72 ℃.

As an additional gene for bacterial identification, the DNA gyrase subunit B (*gyr B)* gene was selected^23,24^. It was used exclusively for identifying bacteria that could not be classified to the species level using the *16S rRNA* gene. Primers UP-1 and UP-2r were used for *gyr B* gene amplification, and primers UP-1S and UP-1Sr were used for Sanger sequencing. The PCR protocol was used as described by Yamamoto and Harayama (1995)^23^.

For fungal identification, PCR amplification of the Internal Transcribed Spacer (ITS) region separated by the 5.8S rRNA gene was amplified using ITS1 and ITS4 primers^25,26^. The PCR protocol consisted of an initial denaturation step of 2 min at 95 °C, followed by 35 cycles of a denaturation step for 45 s at 95 °C, a primer annealing step for 30 s at 55 °C, and an elongation step for 1 min at 72 °C. The final elongation was 4 min at 72 °C. The size of the target fragments varied between 450 and 600 bp.

Due to the inability to identify all fungal representatives using ITS, the *β-tubulin* gene was chosen as an additional marker for fungal identification^27^. Primers Bt2a and Bt2b were used. The amplification protocol included an initiation step for 4 min at 95 °C, followed by 35 cycles of a denaturation step for 45 s at 94 °C, a primer annealing step for 45 s at 58 °C, and an elongation step for 1 min at 72 °C. The final elongation was 6 min at 72 °C. The resulting amplicons were approximately 500 bp in length. The complete list of primers used in the study is shown in Table 1.

**Table 1:** Primers used for PCR amplification and subsequent Sanger sequencing.

Name	Primer sense	Sequence (5′→3′)	Gene	Amplification product (bp)	Reference
27F	Forward	AGAGTTTGATYMTGGCTCAG	*16S rRNA*	∼ 1.400	^21^
1492R	Reverse	GGTTACCTTGTTACGACTT			
UP-1	Forward	GAAGTCATCATGACCGTTCTGCAYGCNGGNGGNAARTTYGA	*gyrB*	∼ 1.100	^23^
UP-2r	Reverse	AGCAGGGTACGGATGTGCGAGCCRTCNACRTCNGCRTCNGTCAT			
UP-1S	Forward	GAAGTCATCATGACCGTTCTGCA			
UP-1Sr	Reverse	AGCAGGGTACGGATGTGCGAGCC			
ITS 1	Forward	TCCGTAGGTGAACCTGCGG	*ITS1* + *5.8S rDNA* + *ITS2*	450–600	^25^
ITS 4	Reverse	TCCTCCGCTTATTGATATGC			
Bt2a	Forward	GGTAACCAAATCGGTGCTGCTTTC	*β-tubulin*	~ 500	^27^
Bt2b	Reverse	ACCCTCAGTGTAGTGACCCTTGGC			

Amplified PCR products were visualised using a gel electrophoresis system (Bio-Rad, Germany) following the addition of SYBR Safe DNA Gel Stain (Thermo Fisher, USA). Subsequent processing was performed only for samples that contained a single target fragment. The clean-up reaction was performed using 0.5 μl Exonuclease I (Thermo Fisher, USA) and 1 μl FastAP Thermosensitive Alkaline Phosphatase (Thermo Fisher, USA) for every 5 μl of unpurified PCR product solution^28^. The resulting mixture was incubated for 15 min at 37 °C, then heated for another 15 min at 85 °C to inactivate enzyme activity in the Thermo Cycler T100 (Bio-Rad, Germany). The concentration of pure amplified fragments was determined using a fluorometer (Qubit Flex, Thermo Fisher, USA) with a Qubit dsDNA BR Assay Kit (Thermo Fisher, USA) according to the manufacturer’s protocol. The purified PCR products were stored in a fridge at 4 ℃.

Sanger sequencing of all samples was performed using Macrogen Europe services (Amsterdam, Netherlands; https://www.macrogen-europe.com/). Before shipping, the concentration was adjusted to 10–20 ng/μl using nuclease-free water (Thermo Fisher, USA) according to the provided recommendations.

### Bioinformatics and statistical data analysis

All ATP and CFU data collected over a study period (Oct-21, Dec-21, Feb-22, Apr-22, May-22, Aug-22, Oct-22), as well as data from the additional sampling in March 2024, were analysed. The Shapiro–Wilk test was used to assess the normality of the distributions of the obtained RLU and CFU data. Calculated W and *p*-value show how closely the data follow a normal distribution. If W is close to 1, the data are normally distributed. If W is around 0.9, some deviated data are present. W < 0.85 indicates strong deviation from normality. *p*-values > 0.05 indicate that the data are normally distributed; *p*-values ≤ 0.05 indicate that the data are not normally distributed.

The median, first quartile (Q1), third quartile (Q3), interquartile range (IQR), and ranges of RLU values and CFU counts were calculated. Total CFU count was calculated for each time point, and a heat map was generated. To assess surface ATP levels, box plots of RLU values for each time point were created for each room. On boxplots, outliers were defined as values exceeding 1.5 times the IQR above Q3 or below Q1.

To assess the strength and direction of association between ATP levels and microbial quantities in WoodW and PaintW rooms separately, non-parametric Spearman’s rank correlation coefficient (ρ) was calculated, and scatter plots were generated. If the ρ coefficient ranges from 0 to 1, it indicates a positive correlation between the two variables. If ρ is 0, there is no correlation, and if ρ varies from − 1 to 0, variables are inversely related^29,30^. All statistical analyses and visualisations were performed in Python 3.14.0 using the *matplotlib, seaborn*, *pandas, scipy* and *numpy* libraries^31–33^.

DNA sequences obtained in this study were compared with the GenBank database at the National Center for Biotechnology Information (NCBI) using the Basic Local Alignment Search Tool (BLAST) software on the NCBI website (http://www.ncbi.nlm.nih.gov/BLAST/) to identify their taxonomic affiliation^34^. The mandatory criteria were sequence coverage > 80%, species-level similarity between sequences of 98–100%, and genus-level similarity of 94–97%. With similarity < 94%, the organism was classified as an unknown fungus or bacterium^35^. The obtained sequences were submitted to GenBank under accession numbers PX917402-PX917429, PX917474-PX917488.

A four-level *Risk Group* (RG) classification was used to identify bacteria and fungi as potential etiological agents of human diseases. These levels are based on the intrinsic virulence of microorganisms and routes of infection^20,36^.

- RG1—bacteria and fungi with low individual and community risk
- RG2—have moderate individual risk and limited community risk, are opportunistic human pathogens.
- RG3—have high individual risk and low community risk and usually cause bacterial diseases or mycoses.
- RG4—bacteria with high individual and community risk usually produce serious human diseases. Not used for fungi.

The RG of all identified bacteria was determined according to the German technical rule TRBA 466^37^, and of fungi according to the information provided in the Atlas of Clinical Fungi^20^.

To estimate the relative abundance of detected bacterial and fungal taxa, the proportion was calculated based on the number of colonies of each identified bacterial/fungal species per Petri dish.

A Venn diagram was constructed to visualise the shared and unique taxonomic units identified in WoodW and PaintW rooms. The presence/absence of each taxonomic unit was taken into account. The diagram was visualized using the *matplotlib-venn* library in Python 3.14.0^38^. In addition, the Jaccard Index was calculated to assess the similarity of microbial community between two locations based on the presence/absence of identified taxonomic units. The index ranges from 0 (completely distinct communities) to 1 (identical communities)^39^. The calculation was performed in Python 3.14.0 using the *pandas* library^33^.

Phylogenetic analysis was performed using the Maximum Likelihood method with 1000 bootstrap replicates in MEGA 12^40^, with preliminary multiple sequence alignment using the ClustalW package^41^. The iTOL tool provided a visualisation of phylogenetic trees^42^.

## Results

### Assessment of surface ATP levels and microbial quantities

A total of 308 measurements were conducted in two patient rooms, comprising 154 ATP and 154 CFU measurements. The mean RLU value for the PaintW room was 414.3, with a measurement range of 5 to 6583. For the WoodW room, the mean RLU was 278.4, with a range of 4 to 1622. Since the ATP threshold for hospital surfaces was set at 100 RLU, 48.7% of measurements in the PaintW room and 64.1% in the WoodW room exceeded this threshold.

**Fig. 3:**
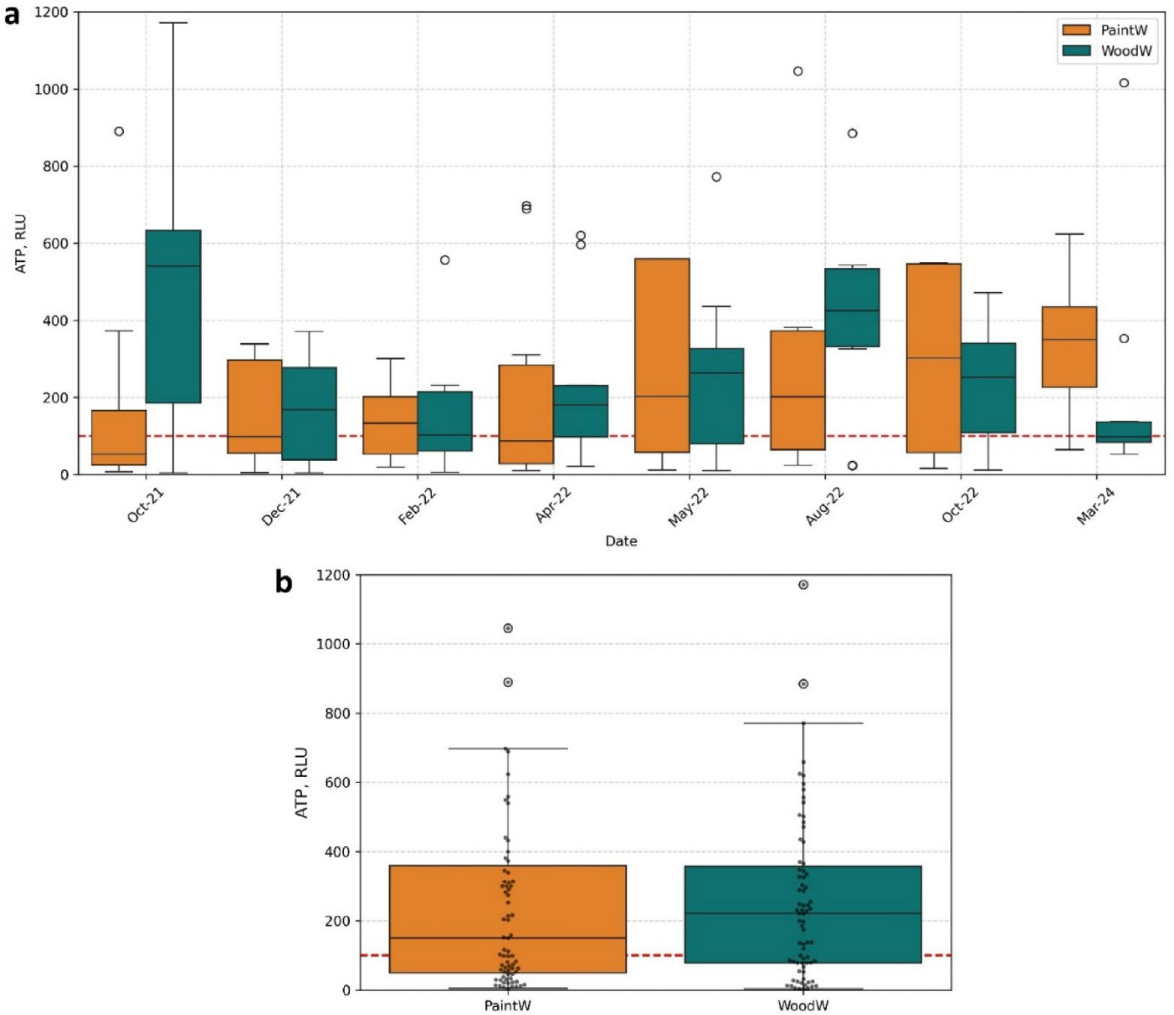
Estimating the amount of ATP on surfaces in patient rooms with painted (PaintW) and wood walls (WoodW). (**a**) Box plot of RLU values detected during 2021–2022 and in March 2024. (**b**) Box plot of all RLU values detected between the PaintW and WoodW patient rooms throughout the study period. The red line indicates the 100 RLU threshold permissible for hospital surfaces. Circles indicate outliers; those > 1200 RLU were excluded from the plots.

Figure 3a demonstrates the distribution of RLU values between the two patient rooms across eight sampling time points during 2021–2022 and in March 2024. The Shapiro–Wilk test indicated that the data significantly deviated from normality (W = 0.41, *p*-value = 3.25 × 10⁻^22^).

The median RLU values for the PaintW room ranged from 53.0 to 350.5 from October 2021 to March 2024. For the WoodW room, median RLU values ranged from 98.0 to 541.0 during the corresponding study period. Of 154 ATP measurements, 18 were identified as outliers. The highest RLU values were 6583 RLU and 4387 RLU on PaintW surfaces in May 2022.

The differences in the distribution of all RLU values measured in both rooms over the entire experimental period are shown in Fig. 3b. The median values were 150.0 RLU for the PaintW room and 225.5 RLU for the WoodW room. No significant statistical difference in RLU distribution was observed between the two patient rooms (*p*-value = 0.1606 (> 0.05)).

**Fig. 4:**
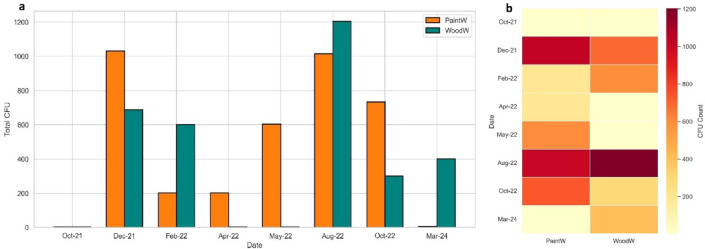
Microbial dynamics on surfaces in patient rooms with painted (PaintW) and wood walls (WoodW). Bar plot (**a**) and heatmap (**b**) with total CFU counts detected during 2021–2022 and in March 2024 are shown.

Simultaneously with ATP measurement, CFU counts were determined on the same surfaces in both patient rooms to assess microbial quantities. The difference between the total CFU values during the period 2021–2022 and March 2024 was shown as a barplot and a heatmap in Fig. 4a and b, respectively.

The total CFU counts in both patient rooms ranged from 2 to 1203. The lowest CFU counts were detected at the beginning of the study in October 2021 in both rooms (3 CFU and 2 CFU), as well as in April and May 2022 in the WoodW room (2 CFU and 2 CFU, respectively), and in March 2024 in the PaintW room (5 CFU). The highest CFU counts were detected for August 2022 in both rooms (1013 CFU and 1203 CFU) and in the PaintW room in December 2021 (1030 CFU).

The Shapiro–Wilk test indicated that CFU data deviated significantly from normality (W = 0.55, *p*-value = 9.27 × 10⁻^20^). Descriptive statistics of CFU counts are shown in Table 2. In particular, the medium CFU counts vary widely, from 0 to 200. The lowest CFU counts were detected in October 2021, April 2022 and March 2024 in both rooms (median 0, IQR 0 and 0.5), in May 2022 in WoodW (median 0, IQR 0), and in February 2022 in PaintW (median 0, IQR 0.5).

**Table 2:** Descriptive statistics of CFU counts detected in PaintW and WoodW patient rooms during 2021–2022 and in March 2024. Non-normal distributed data presented as median (Q1–Q3), Q1, Q3, interquartile range (IQR), and range (minimum–maximum CFU counts).

Date	Location	Median	Q1	Q3	IQR	Range
Oct-21	PaintW	0	0	0	0	0–2
	WoodW	0	0	0	0	0–2
Dec-21	PaintW	104.5	1	200	199	0–215
	WoodW	15	0	200	200	0–230
Feb-22	PaintW	0	0	0.5	0.5	0–200
	WoodW	0	0	200	200	0–200
Apr-22	PaintW	0	0	0	0	0–200
	WoodW	0	0	0	0	0–1
May-22	PaintW	0.5	0	200	200	0–200
	WoodW	0	0	0	0	0–2
Aug-22	PaintW	200	0.5	200	199.5	0–211
	WoodW	200	0.5	200	199.5	0–201
Oct-22	PaintW	22.5	0.5	205.5	205	0–227
	WoodW	0	0	91.5	91.5	0–93
Mar-24	PaintW	0	0	0.5	0.5	0–3
	WoodW	0	0	0.5	0.5	0–200
Total	PaintW	0	0	200	200	0–200
	WoodW	0	0	200	200	0–200

The highest CFU counts were recorded in the PaintW room in December 2021 (median 104.5, IQR 199) and in August 22 in both rooms (median 200, IQR 199.5). Using the benchmark threshold of < 2.5 CFU/cm^2^ (25 CFU per Petri dish), microbial quantifications on hospital surfaces exceeded acceptable levels on half of the sampling dates, except for October 2021, December 2021, August 2022 in both rooms, February 2022 in WoodW, and May 2022 in PaintW.

After evaluating CFU counts across the two patient rooms over the study period, the median and IQR counts in the PaintW and WoodW rooms were equal, and no significant statistical difference was detected (*p* = 0.4293 (> 0.05)).

**Fig. 5:**
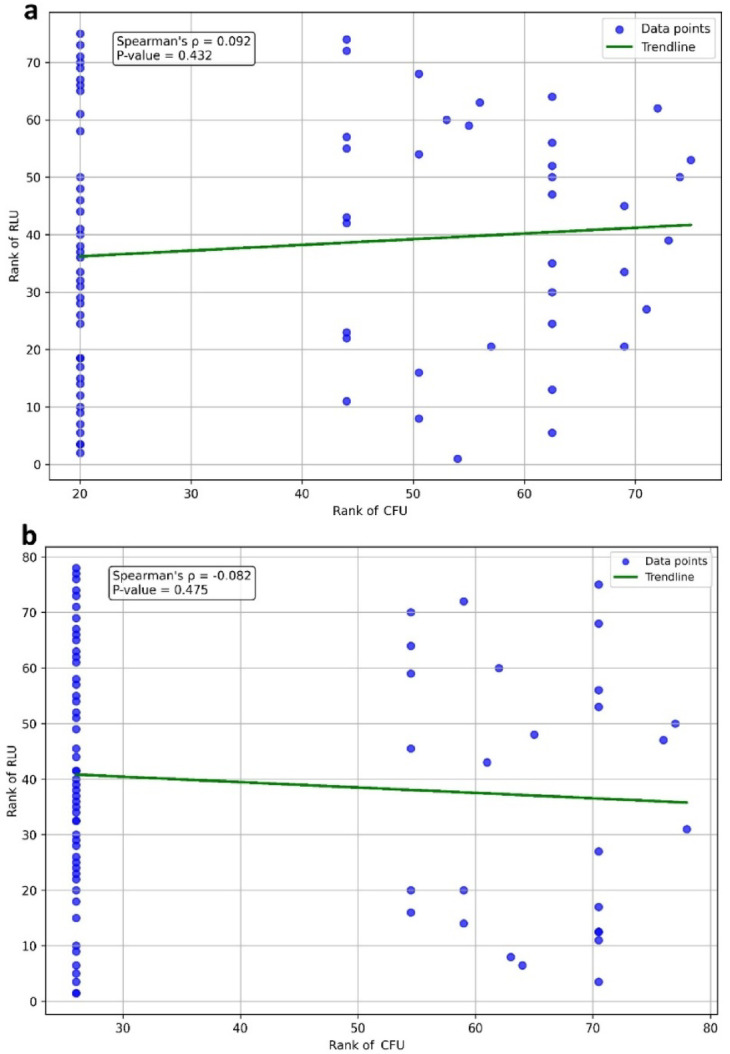
Relationships between the ATP levels and microbial quantities in patient rooms with painted (PaintW) (**a**) and wood walls (WoodW) (**b**) using Spearman’s Rank Correlation. Each RLU value and CFU count was given a rank as the position of a value when all values were ordered from smallest to largest. The obtained RLU and CFU ranks are displayed on the y- and x-axes in scatter plots, respectively. The trendlines (green) show the general direction of correlation between these two variables. In addition, Spearman’s rank correlation coefficient (Spearman’s ρ) and the corresponding *p*-value are shown for data from both rooms.

Spearman’s Rank Correlation was used to assess the relationship between ATP level and microbial quantities in each of the two patient rooms. Figure 5 shows scatterplots with trendlines that visualise the correlation between ATP and CFU measurements across 308 measurements in PaintW (Fig. 5a) and WoodW (Fig. 5b) rooms.

In the PaintW room, a very weak positive correlation between ATP levels and microbial quantities, essentially zero, was observed (Spearman’s coefficient = 0.092). The Spearman’s coefficient for the WoodW room was − 0.082, indicating a very weak negative correlation, essentially close to zero. In both cases, the distributions of ATP and CFU values within each patient’s room were not statistically significant (*p* > 0.05).

### Assessment of microbial diversity

In March 2024, residual microbial presence was evaluated in both patient rooms. A total of 118 pure cultures were isolated and identified by Sanger sequencing, including 75 bacterial and 43 fungal cultures. Of these, 72 microbial pure cultures (48 bacteria and 24 fungi) were isolated from the PaintW patient room, whereas 46 microbial pure cultures (27 bacteria and 19 fungi) were isolated from the WoodW patient room.

By sequencing the full-length *16S rRNA* gene, 48 bacterial cultures were identified to species and 27 to genus. In particular, representatives of the genera *Methylobacterium, Pantoea*, *Micrococcus, Microbacterium, Staphylococcus,* and *Bacillus* were additionally identified by the *gyrB* gene. The *gyrB* gene was used to identify 8 bacterial cultures from the PaintW patient room and 9 from the WoodW patient room. Overall, the *gyrB* gene sequencing enabled species-level identification of bacterial genera *Pantoea*, *Micrococcus*, and *Enterobacter*. At the same time, species-level resolution was not achieved for the *Methylobacterium, Microbacterium, and Bacillus* genera, and amplification of the *gyrB* gene was unsuccessful for isolates of the *Staphylococcus* genus.

ITS sequencing allowed the identification of 32 pure fungal cultures to species and 11 cultures to genus. For *Chaetomium, Aspergillus, Penicillium,* and *Botrytis* genera, species-level identification was insufficient, and additional sequencing using the *β-tubulin* gene was performed. Thus, 9 fungal cultures from the PaintW room and 10 fungal cultures from the WoodW room were additionally identified by the *β-tubulin* gene. This marker gene successfully identified representatives of the *Chaetomium* and *Aspergillus* genera but did not provide sufficient resolution for the *Penicillium* and *Botrytis* genera.

**Fig. 6:**
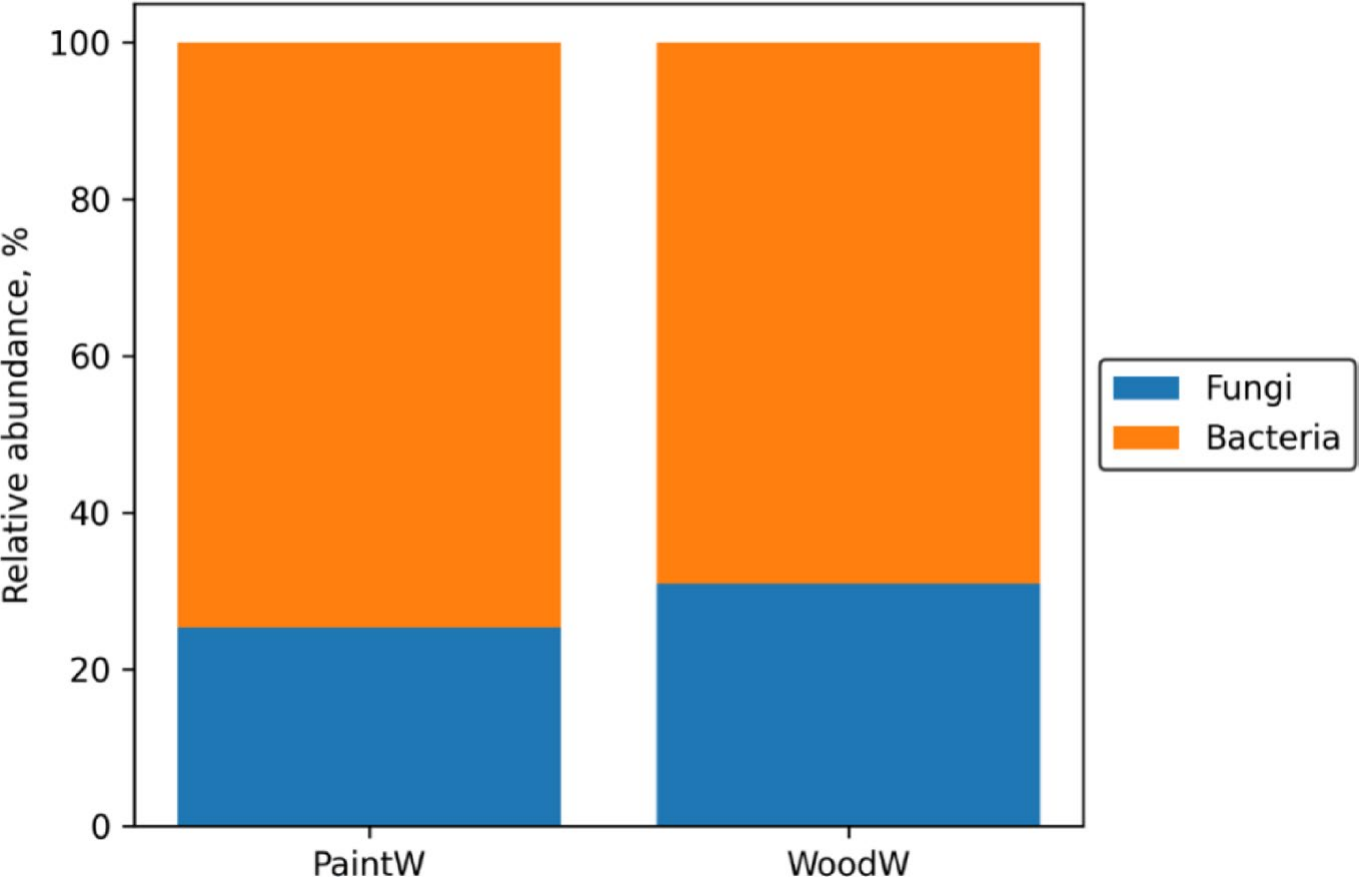
Relative abundance of bacterial and fungal representatives at the domain level on surfaces in patient rooms with painted (PaintW) and wood walls (WoodW).

The relative abundance of bacteria and fungi in the PaintW and WoodW patient rooms is shown in Fig. 6. There were significantly more bacteria than fungi on the surfaces of both patient rooms: 74.6% bacteria and 25.4% fungi in the PaintW room, 68.7% bacteria and 31.3% fungi in the WoodW room.

**Fig. 7:**
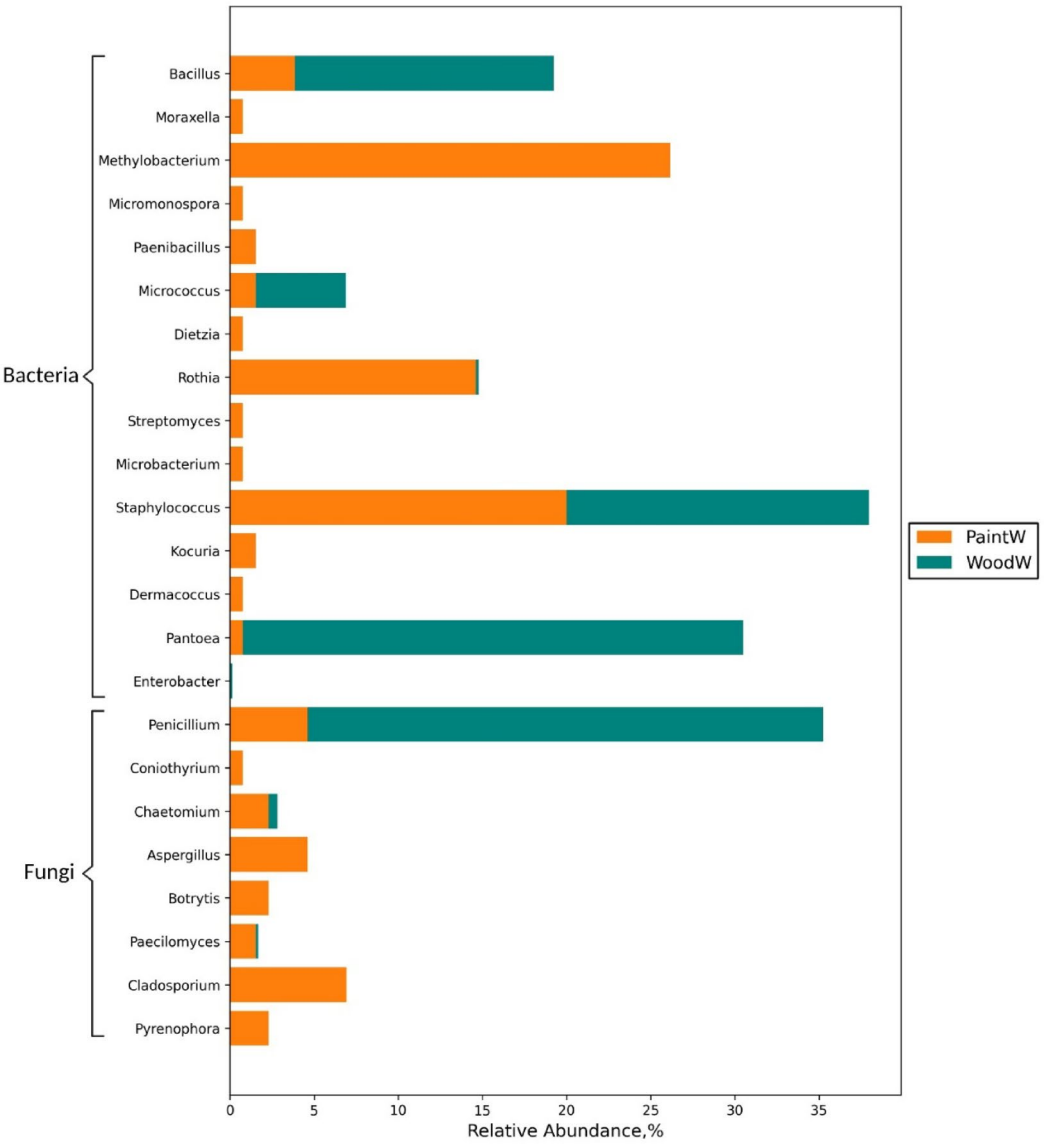
Relative abundance of bacterial and fungal representatives detected on surfaces in patient rooms with painted (PaintW) and wood walls (WoodW) at the genus level.

Overall, 37 species from 23 genera were identified, including 24 bacterial and 13 fungal species. Figure 7 demonstrates the relative abundance of the identified bacterial and fungal genera in both patient rooms.

The bacterial community comprised representatives of 15 genera belonging to the phyla *Bacillota*, *Actinomycetota*, and *Pseudomonadota*. The most frequently detected bacterial genera were *Staphylococcus* (20% in PaintW and 18.1% in WoodW) and *Bacillus* (3% in PaintW and 15.5% in WoodW). Additionally, 26.2% and 14,6% of the bacterial community in the PaintW room belonged to the genera *Methylobacterium* and *Rothia*, respectively. The most abundant bacterial genus in the WoodW room was *Pantoea*, which accounted for approximately 29.7% of the microbial community.

All identified fungi belonged to 8 genera of the *Ascomycota* phylum. In the PaintW room, approximately 1% to 7% of the microbial community was comprised of fungi from all 8 genera. All fungi found in the WoodW room belonged to the genera *Penicillium* (30.8%), *Chaetomium* (0.5%), and *Paecilomyces* (0.15%).

Among the 24 identified bacterial species, both mesophilic aerobic and anaerobic species were found according to information available in the databases BacDive and NCBI. Most bacterial representatives were in risk group 1 (RG1). At the same time, *Bacillus cereus, Moraxella osloensis, Pantoea septica*, *Staphylococcus epidermidis*, and *Staphylococcus hominis* were assigned to RG2 in PaintW. In the WoodW room, *Enterobacter asburiae, P. septica*, and *S. hominis* belonged RG2. *Bacillus* sp. and *Staphylococcus* sp., which were present in the microbial communities of both rooms, were considered to potentially belong to RG 1 or RG 2; however, species-level assignment was not possible. The vast majority of identified fungi belonged to saprotrophs and moulds, and were classified as RG1. No representatives of RG3 and RG4, which pose a high risk to humans, were identified. Detailed information on all bacterial and fungal species identified in the study is shown in Tables 3 and 4.

**Table 3:** Bacteria species identified on surfaces in patient rooms with painted (PaintW) and wood walls (WoodW), with their relative abundance, Risk group, and marker genes used for identification.

Species	Phylum^1^	Live style^1^	Relative abundance %		Risk Group (RG)^2^	Marker gene
			PaintW	WoodW		
*Bacillus cereus* Frankland and Frankland	Bacillota	anaerobe, mesophilic bacteria, opportunistic human pathogen	0.8	–	2	16S rRNA, *gyrB*
*Bacillus* sp.	Bacillota	aerobe or anaerobe, mesophilic bacteria	3.1	14.9	1–2	16S rRNA, *gyrB*
*Bacillus subtilis* (Ehrenberg) Cohn	Bacillota	anaerobe, mesophilic bacteria, opportunistic human pathogen	–	0.2	1	16S rRNA
*Bacillus velezensis* Ruiz-García	Bacillota	aerobe, mesophilic bacteria	–	0.2	1	16S rRNA
*Dermacoccus nishinomiyaensis (Oda) Stackebrandt*	Actinomycetota	mesophilic bacteria, opportunistic human pathogen	0.8	–	1	16S rRNA
*Dietzia cinnamea* (Nesterenko and Harrison) Rainey	Actinomycetota	microaerophile, mesophilic bacteria, opportunistic human pathogen	0.8	–	1	16S rRNA
*Enterobacter asburiae* Brenner	Pseudomonadota	anaerobe, mesophilic bacteria, opportunistic human pathogen	–	0.1	2	16S rRNA*, gyrB*
*Kocuria marina* Kim	Actinomycetota	aerobe, mesophilic bacteria, opportunistic human pathogen	1.5	–	1	16S rRNA
*Methylobacterium adhaesivum* Gallego	Pseudomonadota	aerobic, mesophilic bacteria	2.3	–	1	16S rRNA
*Methylobacterium* sp.	Pseudomonadota	aerobic, mesophilic bacteria	23.8	–	1	16S rRNA, gyrB
*Microbacterium* sp.	Actinomycetota	aerobic, heat-resistant bacteria	0.8	–	1	16S rRNA, gyrB
*Micrococcus luteus* (Schroeter) Cohn	Actinomycetota	aerobe, mesophilic bacteria	1.5	5.3	1	16S rRNA, gyrB
*Micromonospora aurantiaca* Sveshnikova	Actinomycetota	mesophilic bacteria	0.8	–	1	16S rRNA
*Moraxella osloensis* Bøvre and Henriksen	Pseudomonadota	aerobe, mesophilic bacteria, symbiont of nematode *Phasmarhabditis hermaphrodita* Schneider	0.8	–	2	16S rRNA
*Paenibacillus* sp.	Bacillota	anaerobic, mesophilic bacteria	1.5	–	1	16S rRNA
*Pantoea septica* Brady	Pseudomonadota	aerobe, mesophilic bacteria, opportunistic human pathogen	0.8	29.7	2	16S rRNA, gyrB
*Rothia amarae Fan*	Actinomycetota	microaerophile, mesophilic bacteria, opportunistic human pathogen	–	0.1	1	16S rRNA
*Rothia kristinae* (Kloos) Nouioui	Actinomycetota	microaerophile, mesophilic bacteria, opportunistic human pathogen	14.6	0.1	1	16S rRNA
*Staphylococcus capitis* Kloos and Schleifer	Bacillota	aerobe, mesophilic bacteria, opportunistic human pathogen	5.4	–	1	16S rRNA
Staphylococcus epidermidis (Winslow and Winslow) Evans	Bacillota	anaerobe, mesophilic bacteria, human pathogen	6.9	–	2	16S rRNA
*Staphylococcus hominis* Kloos and Schleifer	Bacillota	aerobe, mesophilic bacteria, opportunistic human pathogen	3.1	17.8	2	16S rRNA
*Staphylococcus* sp.	Bacillota	aerobe or anaerobe, mesophilic bacteria, human pathogen	4.6	0.1	1–2	16S rRNA
*Staphylococcus warner*i Kloos and Schleifer	Bacillota	aerobe, mesophilic bacteria, opportunistic human pathogen	–	0.1	2	16S rRNA
*Streptomyces thermocarboxydus* Kim	Actinomycetota	aerobe, thermophilic bacteria	0.8	–	1	16S rRNA

^1^Data taken from databases BacDive (https://beta.bacdive.dsmz.de/), NCBI Taxonomy Browser (https://www.ncbi.nlm.nih.gov/).

^2^Risk groups for bacteria are defined according to TRBA 466 technical rule^36^.

**Table 4:** Fungal species identified on surfaces in patient rooms with painted (PaintW) and wood walls (WoodW), with their relative abundance, Risk group, and marker genes used for identification.

Species	Phylum^1^	Live style^1^	Relative abundance %		Risk Group^2^	Marker gene
			PaintW	WoodW		
*Aspergillus niger* van Tieghem	Ascomycota	Saprotroph, mould, opportunistic human pathogen	2.3	–	1	ITS, β-tubulin
*Aspergillus tubingensis *Mosseray	Ascomycota	Saprotroph, mould, opportunistic human pathogen	2.3	–	1	ITS, β-tubulin
*Botrytis* sp.	Ascomycota	Saprotroph, mould	2.3	–	Unknown	ITS, β-tubulin
*Chaetomium globosum* Kunze	Ascomycota	Saprotroph, mould, opportunistic human pathogen	2.3	0,5	1	ITS, β-tubulin
*Cladosporium herbarum* (Pers.) Link	Ascomycota	Saprotroph	0.8	–	1	ITS, β-tubulin
*Cladosporium halotolerans* Zalar, de Hoog, Schroers, Crous, Groenewald and Gunde-Cimerman	Ascomycota	Saprotroph	6.2	–	1	ITS
*Coniothyrium telephii* (Allescher) Verkley and Gruyter	Ascomycota	Saprotroph, opportunistic human pathogen	0.8	–	1	ITS
*Paecilomyces variotii* Bainier	Ascomycota	Saprotroph, opportunistic human pathogen	1.5	0.1	1	ITS
*Penicillium chrysogenum* Thom	Ascomycota	Saprotroph, mould	2.3	–	1	ITS, β-tubulin
*Penicillium corylophilum*	Ascomycota	Saprotroph, mould	2.3	0.1	1	ITS
*Penicillium digitatum* (Persoon) Saccardo	Ascomycota	Saprotroph, mould	–	0.1	1	ITS
*Penicillium* sp.	Ascomycota	Saprotroph, mould	–	30.4	1	ITS, β-tubulin
*Pyrenophora triseptata* (Drechsler) Rossman and Hyde	Ascomycota	Plant pathogen	2.3	–	1	ITS

^1^NCBI Taxonomy Browser (https://www.ncbi.nlm.nih.gov/), and the Atlas of Clinical Fungi^20^.

^2^Risk groups for bacteria are defined according to the Atlas of Clinical Fungi^20^.

**Fig. 8:**
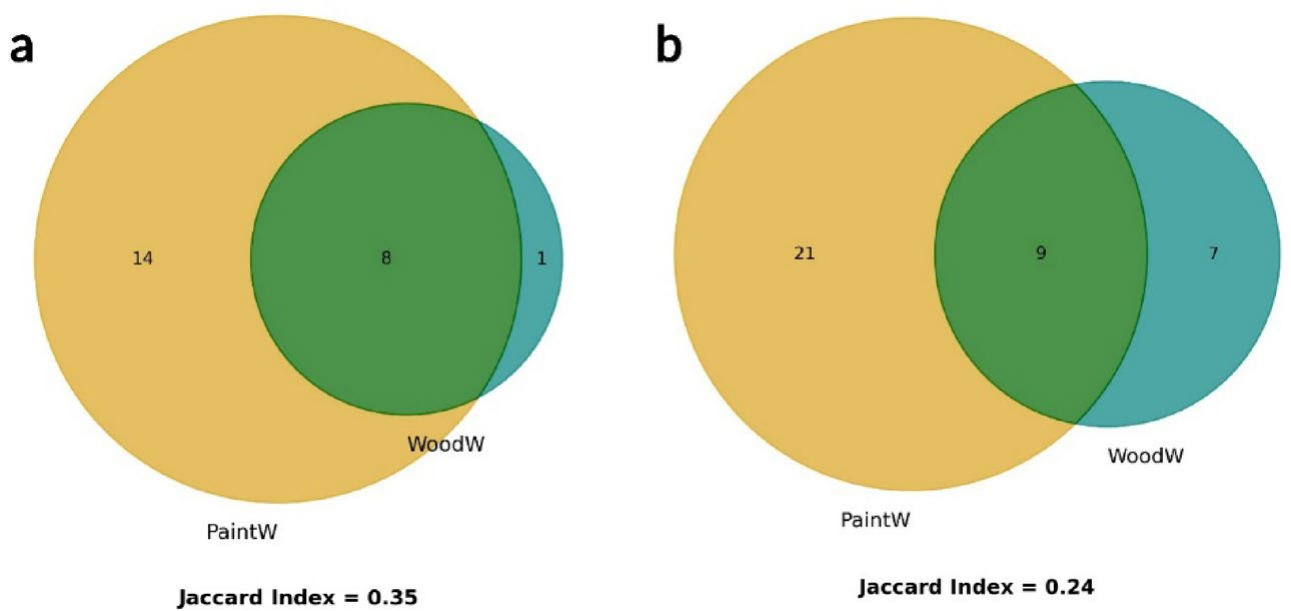
Venn diagrams illustrate the unique and shared genera (**a**) and species (**b**) of fungi and bacteria identified in patient rooms with painted (PaintW) and wood walls (WoodW). The yellow circle represents unique taxa from the PaintW room, the blue circle represents unique taxa from the WoodW room. The green overlap area shows the number of shared taxa. The Jaccard Index (below the diagrams) illustrates the level of similarity between the microbial communities in the two rooms at the genus (**a**) and species (**b**) levels.

To assess the similarity of microbial communities between the two rooms, Venn diagrams were constructed to visualise the numbers of unique and shared fungal and bacterial taxa between the PaintW and WoodW patient rooms. Figure 8a shows that 14 unique genera were identified in PainW room and 1 genus in WoodW room, while eight genera were shared between rooms (*Paecilomyces*, *Penicillium*, *Chaetomium, Rothia*, *Staphylococcus*, *Pantoea*, *Micrococcus*, and *Bacillus)*. The corresponding Jaccard Index was 0.35.

The diagram in Fig. 8b shows the presence of 21 unique species in the PaintW room and 7 unique species in the WoodW room. 9 shared species were identified in both patient rooms (*Paecilomyces variotii*, *Penicillium corylophilum*, *Chaetomium globosum*, *Rothia kristinae*, *S. hominis, Staphylococcus sp*., *P. septica*, *Micrococcus luteus*, and *Bacillus* sp.). The Jaccard Index was 0.24. Overall, the data obtained demonstrates low similarity in microbial species and genus composition between the two patient rooms studied.

**Fig. 9:**
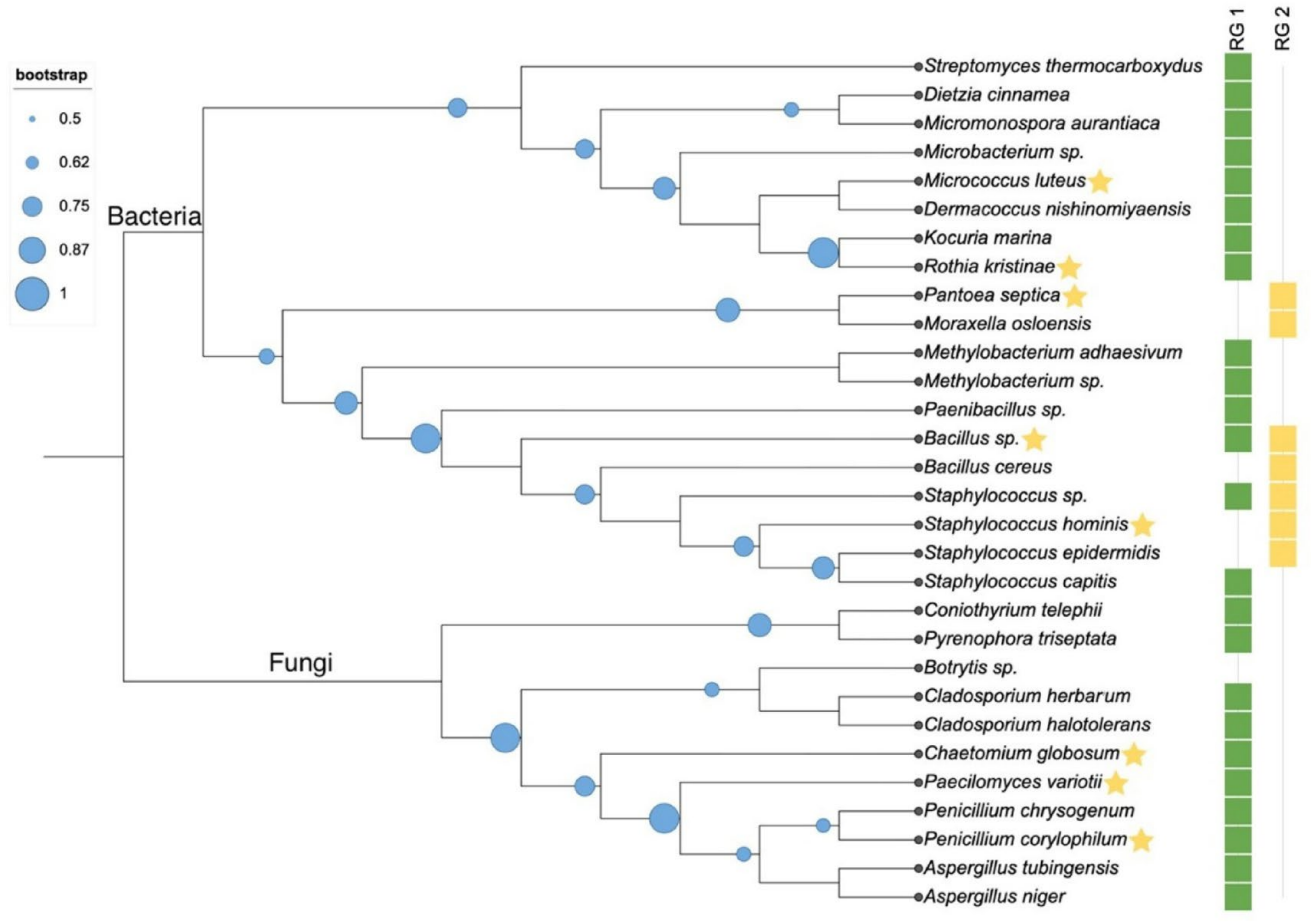
Maximum Likelihood phylogenetic tree showing the taxonomic position of all identified bacterial and fungal species isolated from the surfaces in patient rooms with painted (PaintW) based on multiple alignments of *16S rRNA* and ITS sequences. Bootstrap values (> 50%) are indicated on branches as blue circles. Species detected in the WoodW patient room are also marked with a yellow star. The green or yellow rectangles (on the right side) indicate risk groups (RG) of each species.

The phylogenetic relationships among the identified bacterial and fungal species were assessed based on the obtained 16S rRNA and ITS sequences. Figure 9 shows a phylogenetic tree containing the 30 microbial species from the PaintW patient room. A clear clustering of bacterial species was observed, distinct from that of fungal species. Bacteria belonging to the phylum *Actinomycetota* formed a separate clade, which included *Streptomyces thermocarboxydus*, *Dietzia cinnamea*, *Micromonospora aurantiaca*, *Microbacterium* sp., *Micrococcus luteus*, *Dermacoccus nishinomiyaensis*, *Rothia kristinae*, and *Kocuria marina*. Species of the phylum *Bacillota* (*Paenibacillus*, *Bacillus,* and *Staphylococcus* sp.) clustered together with a 100% bootstrap rate.

**Fig. 10:**
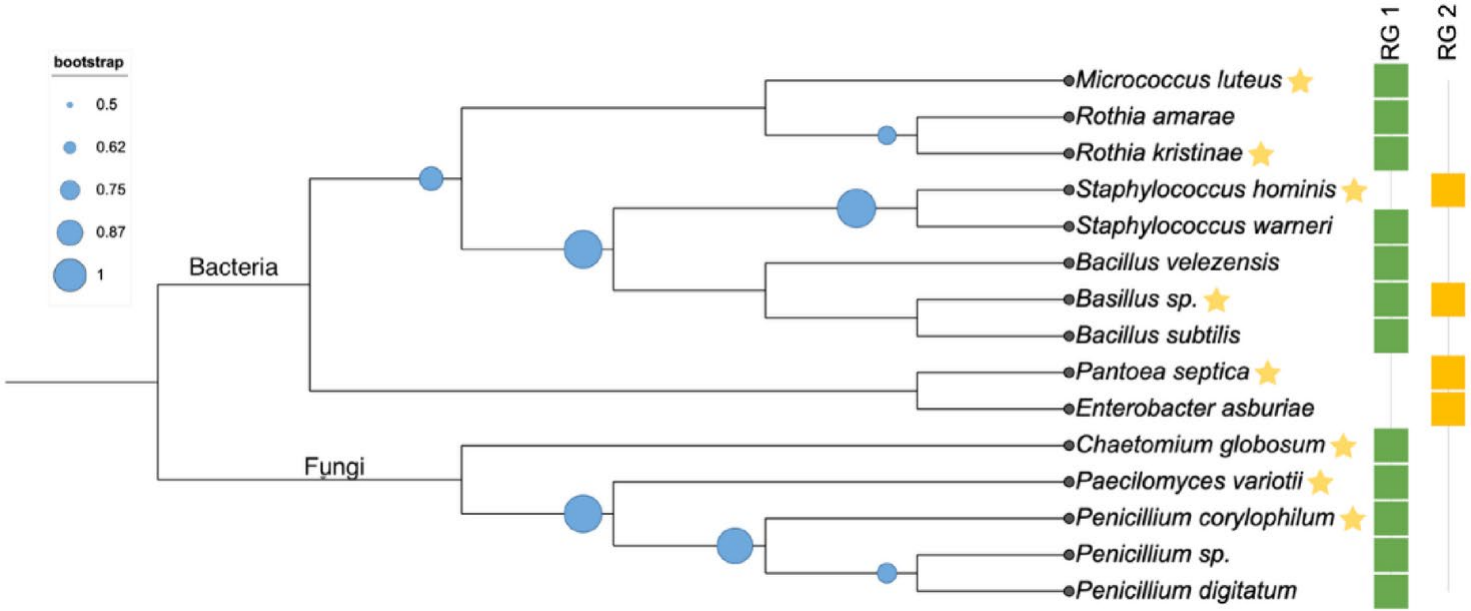
Maximum Likelihood phylogenetic tree showing the taxonomic position of all identified bacterial and fungal species isolated from the surfaces in patient rooms with wood wall (WoodW) based on multiple alignments of *16S rRNA* and ITS sequences. Bootstrap values (> 50%) are indicated on branches as blue circles. Species detected in the PaintW patient room are also marked with a yellow star. The green or yellow rectangles (on the right side) indicate risk groups (RG) of each species.

The phylogenetic tree, which contains 15 species isolated from surfaces in the WoodW patient room, is shown in Fig. 10. Separate clades were formed for bacterial and fungal species. Additionally, a clear separation of bacterial species by phylum affiliation was observed. *P. septica* and *E. asburiae* formed a separate clade as representatives of the *Pseudomonadota*, *M. luteus* and *Rothia* species—as *Actinomycetota*, and *Bacillus* and *Staphylococcus* species—as *Bacillota*. In addition, both phylogenetic trees show clear groupings within the same genus, particularly *Staphylococcus*, *Bacillus*, *Methylobacterium*, *Penicillium*, *Aspergillus*, and others.

## Discussion

Improving the hospital environment, particularly through interior changes, positively affects the well-being and comfort of both patients and medical staff^3,43^. However, significant changes in patients’ stay conditions, particularly the installation of new materials in patient rooms, such as wood panels, can affect cleanliness, environmental contamination, and the biodiversity of the microbial community within those facilities. The study of such an impact is a crucial step towards the widespread adoption of wooden materials for the decoration and design of hospital premises.

This study assessed the microbial dynamics and residual diversity on surfaces in two patient rooms in the same hospital ward. The main difference between the patient rooms was the presence of designer wood panels. Measurements of ATP levels and microbial quantities indicated that surface contamination fluctuated over time in both rooms, reflecting the dynamic nature of hospital environments. Although a substantial proportion of ATP measurements exceeded the benchmark threshold of 100 RLU, no statistically significant differences in ATP levels were observed between the room with painted walls and the room with wooden panels. This suggests that no statistically significant differences in overall surface cleanliness were detected between the two patient rooms under the conditions investigated.

During the study period, high microbial levels were observed in both rooms, frequently exceeding the recommended thresholds for hospital surfaces^16,18^. However, these findings indicate that no statistically significant differences in microbial quantities were detected between the two rooms during the study period. Because only one room per wall type was available, these observations should not be interpreted as demonstrating a causal effect of wall material.

The discussion about the relationship between ATP levels and microbial quantities remains relevant today. Consistent with several previous studies^12,44–47^, no meaningful correlation between ATP levels and microbial quantities on surfaces in both patient rooms. This lack of correlation reflects the fact that ATP bioluminescence detects total organic material on surfaces, including both microbial and non-microbial residues, such as skin flakes, body fluids, and food particles. On the other hand, even if microbial quantities are low, the presence of other biological material on surfaces can serve as a source of nutrients for bacterial and fungal growth, including pathogens^12^. While ATP monitoring remains a fast tool for assessing overall surface cleanliness, it cannot be used as a direct indicator of microbial load or sterility. Therefore, the removal of organic residues remains important, as such material may serve as a substrate for microbial growth^46^.

This study highlights differences in microbial diversity between patient room surfaces with different wall materials. Molecular identification of the isolates revealed substantially greater diversity of bacterial and fungal species on surfaces in the painted-wall room compared with the room with wooden wall panels. At both the genus and species levels, microbial community similarity between the two rooms was low, suggesting distinct surface-associated microbiomes. Previous studies showed that the formation of the surface microbiome in hospitals is a complex dynamic process influenced by a number of factors, including the effects of surface materials, clinical activities^7^, cleaning and ventilation schedules, and the number of patients who actively release their own microbes into hospital wards, thus changing the environment^48,49^. Because the patient rooms were located in the same ward and shared similar characteristics, cleaning regimens, ventilation conditions, and clinical functions, wall materials may have contributed to the observed differences. However, room-specific characteristics cannot be entirely ruled out, and the observational design does not allow the effect of wall material to be distinguished unequivocally from other room-level factors.

Lower microbial diversity was observed in the room with wooden wall panels. However, because only one room was used for each wall type, this observation should be interpreted with caution until confirmed in replicated studies. One possible explanation for the lower microbial diversity observed in the room with wooden wall panels relates to the inherent properties of wood. Previous studies have suggested that wood may exhibit antimicrobial effects due to its porous, hygroscopic structure and chemical composition^50,51^. By absorbing moisture from the surface, wood may reduce microbial survival through desiccation^9,50,51^, while certain wood extractives have been reported to possess antimicrobial activity^52–54^. Although these mechanisms are consistent with the observed patterns, it is also worth considering a fire-retardant coating on wood panels as such a factor, which potentially can influence microbial diversity, but is mandatory for the use of wood in interior indoor applications. So the present study was not designed to directly test antimicrobial activity, and alternative explanations, for instance, microclimatic differences at the surface level, cannot be excluded, and pattern explanation requires detailed future study.

The culture-based approach used in this study allowed detailed identification of viable bacterial and fungal representatives but also imposed limitations on species resolution and community coverage compared with DNA-based approaches. While 16S rRNA gene sequencing and ITS sequencing enabled identification of most isolates, several bacterial and fungal genera required additional markers, and some taxa could not be resolved to the species level. Future studies incorporating complementary molecular approaches, such as high-throughput sequencing, could provide a more comprehensive view of microbial community composition and functional potential.

Given that the wood-panelled patient room is the only one to date, an important limitation of this study is the lack of room-level replicates. Although multiple sampling points and repeated measurements over time were included, these are repeated observations within a single experimental unit and not independent biological replicates. Therefore, patient room-specific characteristics cannot be completely separated from the effects of wall material. Therefore, the results obtained should be interpreted as observational evidence of an association rather than as proof of a causal effect. Future studies involving multiple replicate patient rooms in different hospitals are needed to confirm these observations.

In addition to the limited room-level replication, the observational nature of the study did not allow control over all variables that may influence the hospital surface microbiome. Patient occupancy, patient turnover, diagnoses, visitor frequency, and daily clinical activities inevitably varied during routine hospital operation and could have contributed to temporal and spatial variation in microbial contamination and diversity. Although selecting two highly comparable rooms reduced environmental variability, residual confounding cannot be excluded.

Overall, the results demonstrate that installing wooden wall panels in a hospital patient room did not compromise surface microbial dynamics. At the same time, the presence of wood was associated with a distinct, less diverse surface microbiome than in a painted-wall room. Our findings support the careful consideration of wood as an interior material in hospitals with appropriate cleaning and maintenance. Further long-term, multi-site studies are needed to better understand the formation of the surface microbiome and to evaluate the broader implications of wood use in healthcare facilities.

## Conclusion

In this study, surface microbial dynamics and residual diversity in two patient rooms with painted and wooden walls were assessed, and the microbial biodiversity in these rooms was investigated. High ATP levels and microbial quantities were detected on the surfaces of both patient rooms at half-time points, indicating abnormal surface contamination and insufficient cleanliness. Importantly, no statistically significant differences in ATP levels or microbial quantities were observed between the two patient rooms throughout the study period. No clear correlation was found between ATP levels and microbial quantities in both patient rooms, because the ATP method measures total organic material on surfaces rather than viable microorganisms alone. This highlights the complementary roles of ATP monitoring and culture-based methods in evaluating hospital surface hygiene.

Culture isolation and Sanger sequencing revealed that all bacterial and fungal species belonged exclusively to risk groups 1 and 2, indicating a low risk to human health. Microbial biodiversity differed significantly between the rooms, with a higher number of bacterial and fungal species detected in the painted-wall room, whereas the presence of wooden wall panels was associated with lower microbial biodiversity without increased contamination. This pattern may be related to intrinsic properties of wood, such as its physical structure or chemical composition, which confer antimicrobial properties; further targeted studies of these phenomena are required.

Overall, the room with wood wall panels was not associated with significantly higher levels of microbial contamination than the control room with painted walls. However, because this study included only one room per wall type, the results should be interpreted with caution and not considered definitive evidence of the effect of wall material. Further studies involving replicate patient rooms across different healthcare facilities with control of several operational factors are needed to confirm this, but these results support careful consideration of wood in hospital facility design, along with appropriate cleaning and maintenance practices.

## Data Availability

The datasets generated and analysed during the current study are available in GenBank database at NCBI, accession numbers PX917402-PX917429, PX917474-PX917488.

## References

[1] Mauri, D. et al. Interior design: A new perspective in supportive care of patients with acute onset of debilitating diseases. *Palliat. Med. Rep.* **2**, 365–368 (2021). 35983237 10.1089/pmr.2021.0031 PMC9380877

[2] Foster, M. W. et al. The effects of viewing visual artwork on patients, staff, and visitors in healthcare settings: A scoping review. *PLoS ONE* **20**, e0328215. 10.1371/journal.pone.0328215 (2025). 40833932 10.1371/journal.pone.0328215 PMC12367177

[3] Alhsainat, A. & Günçe, K. Healing environment in pediatric cancer centers by utilizing positive distractions. *Behav. Sci.* **14**, 1010. 10.3390/bs14111010 (2024). 39594310 10.3390/bs14111010 PMC11591362

[4] Minunno, R., O’Grady, T., Morrison, G. M. & Gruner, R. L. Investigating the embodied energy and carbon of buildings: A systematic literature review and meta-analysis of life cycle assessments. *Renew. Sustain. Energy Rev.* **143**, 110935. j.rser.2021.110935 (2021).

[5] UNECE. Carbon storage in harvested wood products (HWP). UNECE. Available at: https://unece.org/forests/carbon-storage-harvested-wood-products-hwp (accessed 9 April 2021).

[6] Hill, C. A. S. & Dibdiakova, J. The environmental impact of wood compared to other building materials. *Int. Wood Prod. J.* **7**, 215–219 (2016).

[7] Chibwe, K. et al. Intra-hospital microbiome variability is driven by accessibility and clinical activities. *Microbiol. Spectr.* **12**, e00296-e324. 10.1128/spectrum.00296-24 (2024). 38940596 10.1128/spectrum.00296-24 PMC11302010

[8] Gilbert, J. A. & Stephens, B. Microbiology of the built environment. *Nat. Rev. Microbiol.* **16**, 661–670 (2018). 30127345 10.1038/s41579-018-0065-5

[9] Kettunen, E. et al. Touch the wood: Antimicrobial properties of wooden and other solid material surfaces differ between dry and moist contamination in public and laboratory exposure. *Environ. Adv.* **13**, 100416. 10.1016/j.envadv.2023.100416 (2023).

[10] Munir, M. T. et al. Hygienic perspectives of wood in healthcare buildings. *Hygiene* **1**, 12–23 (2021).

[11] Giovinazzo, R. et al. Benchmark guidance values for microbiological monitoring on surfaces: A literature overview. *Biomed. Prev. Issues* **4**, 135. 10.19252/000000087 (2017).

[12] Arkel, A., Willemsen, I. & Kluytmans, J. The correlation between ATP measurement and microbial contamination of inanimate surfaces. *Antimicrob. Resist. Infect. Control* **10**, 116. 10.1186/s13756-021-00981-0 (2021). 34362450 10.1186/s13756-021-00981-0 PMC8349058

[13] Sherlock, O., O’Connell, N., Creamer, E. & Humphreys, H. Is it really clean? An evaluation of the efficacy of four methods for determining hospital cleanliness. *J. Hosp. Infect.* **72**(2), 140–146 (2009). 19321226 10.1016/j.jhin.2009.02.013

[14] Svensk Förening för Vårdhygien. BOV—Byggenskap och vårdhygien (2016).

[15] Amodio, E. et al. Analytical performance issues: Comparison of ATP bioluminescence and aerobic bacterial count for evaluating surface cleanliness in an Italian hospital. *J. Occup. Environ. Hyg.* **11**(2), D23–D27 (2014). 24369935 10.1080/15459624.2013.852281 PMC7196686

[16] Mulvey, D. et al. Finding a benchmark for monitoring hospital cleanliness. *J. Hosp. Infect.* **77**, 25–30 (2011). 21129820 10.1016/j.jhin.2010.08.006

[17] SIS Swedish Institute for Standards. SS-ISO 16000-21:2013 (2015) Indoor air—Part 21: Detection and enumeration of moulds—Sampling from materials. Available at https://www.sis.se/produkter/miljo-och-halsoskydd-sakerhet/miljoskydd/nedsmutsning-regler-och-skydd/ssiso16000212015/.

[18] Dancer, S. J. Hospital cleaning in the 21st century. *Eur. J. Clin. Microbiol. Infect. Dis.* **30**, 1473–1481 (2011). 21499954 10.1007/s10096-011-1250-x

[19] Gerrits van den Ende, A. H. G. & de Hoog, G. S. Variability and molecular diagnostics of the neurotropic species *Cladophialophora bantiana*. *Stud. Mycol.* **43**, 151–162 (1999).

[20] de Hoog, G. S. et al. *Atlas of clinical fungi: The ultimate benchtool for diagnostics*, 4th edition. (Fondation Atlas of Clinical Fungi, Hilversum, Netherlands, 2020).

[21] Lane, D. J. 16S/23S rRNA sequencing. In *Nucleic Acid Techniques in Bacterial Systematics* 115–175 (John Wiley & Sons, 1991).

[22] Eden, P. A., Schmidt, T. M., Blakemore, R. P. & Pace, N. R. Phylogenetic analysis of *Aquaspirillum magnetotacticum* using polymerase chain reaction amplified 16S rRNA-specific DNA. *Int. J. Syst. Bacteriol.* **41**, 324–325 (1991). 1854644 10.1099/00207713-41-2-324

[23] Yamamoto, S. & Harayama, S. PCR amplification and direct sequencing of *gyrB* genes with universal primers and their application to the detection and taxonomic analysis of *Pseudomonas putida* strains. *Appl. Environ. Microbiol.* **61**, 1104–1109 (1995). 7793912 10.1128/aem.61.3.1104-1109.1995 PMC167365

[24] Hannula, M. & Hänninen, M.-L. Phylogenetic analysis of *Helicobacter* species based on partial *gyrB* gene sequence. *Int. J. Syst. Evol. Microbiol.* **57**, 444–449 (2007). 17329766 10.1099/ijs.0.64462-0

[25] White, T. J., Bruns, T. D., Lee, S. B. & Taylor, J. W. Amplification and direct sequencing of fungal ribosomal RNA genes for phylogenetics. In *PCR Protocols: A Guide to Methods and Applications* (eds Innis, M. A. et al.) 315–322 (Academic Press, 1990).

[26] Bellemain, E. et al. ITS as an environmental DNA barcode for fungi: An *in silico* approach reveals potential PCR biases. *BMC Microbiol.* **10**, 189 (2010). 20618939 10.1186/1471-2180-10-189 PMC2909996

[27] Glass, N. L. & Donaldson, G. C. Development of primer sets designed for use with the PCR to amplify conserved genes from filamentous ascomycetes. *Appl. Environ. Microbiol.* **61**, 1323–1330 (1995). 7747954 10.1128/aem.61.4.1323-1330.1995 PMC167388

[28] Werle, E., Schneider, C., Renner, M., Völker, M. & Fiehn, W. Convenient single-step, one-tube purification of PCR products for direct sequencing. *Nucleic Acids Res.* **22**(20), 4354–4355 (1994). 7937169 10.1093/nar/22.20.4354 PMC331970

[29] Boddy, R. & Smith, G. *Statistical Methods in Practice: For Scientists and Technologists* 95–96 (Wiley, 2009).

[30] Akoglu, H. User’s guide to correlation coefficients. *Turk. J. Emerg. Med.* **18**(3), 91–93 (2018). 30191186 10.1016/j.tjem.2018.08.001 PMC6107969

[31] Han, S. & Kwak, I. Y. Mastering data visualization with Python: Practical tips for researchers. *J. Minim. Invasive Surg.* **26**(4), 167–175 (2023). 38098348 10.7602/jmis.2023.26.4.167 PMC10728683

[32] Stancin, I. & Jovic, A. An overview and comparison of free Python libraries for data mining and big data analysis. In *42nd Int. Conv. Inf. Commun. Technol. Electron. Microelectron. MIPRO 2019—Proc.* 977–982 (2019).

[33] Molin, S. *Hands-On Data Analysis with Pandas: Efficiently Perform Data Collection, Wrangling, Analysis, and Visualization Using Python* (Packt Publishing, 2019).

[34] Ye, J., McGinnis, S. & Madden, T. L. BLAST: Improvements for better sequence analysis. *Nucleic Acids Res.* **34**, W6–W9 (2006). 16845079 10.1093/nar/gkl164 PMC1538791

[35] Davydenko, K. et al. Fungal pathogens associated with *Tomicus* species in European forests: Regional variations and impacts on forest health. *Insects* **16**, 277. 10.3390/insects16030277 (2025). 40266754 10.3390/insects16030277 PMC11943276

[36] TRBA. TRBA 450: Classification criteria for biological agents. *GMBl* **23**, 1–18 (22 June 2016)

[37] TRBA. TRBA 466: Classification of prokaryotes (Bacteria and Archaea) into risk groups. *GMBl* **68–80**, 1–254 (2010).

[38] Tretyakov, K. Matplotlib-Venn—functions for plotting area-proportional two- and three-way Venn diagrams in matplotlib, Python Package Version 0.11.6 (2020). Available at https://pypi.org/project/matplotlib-venn/.

[39] Chung, N. C., Miasojedow, B., Startek, M. & Gambin, A. Jaccard/Tanimoto similarity test and estimation methods for biological presence-absence data. *BMC Bioinform.* **20**, 644. 10.1186/s12859-019-3118-5 (2019). 10.1186/s12859-019-3118-5 PMC6929325 31874610

[40] Kumar, S. et al. MEGA12: Molecular evolutionary genetic analysis version 12 for adaptive and green computing. *Mol. Biol. Evol.* **41**(12), msae263. 10.1093/molbev/msae263 (2024). 39708372 10.1093/molbev/msae263 PMC11683415

[41] Thompson, J. D., Higgins, D. G. & Gibson, T. J. CLUSTAL W: Improving the sensitivity of progressive multiple sequence alignment through sequence weighting, position-specific gap penalties and weight matrix choice. *Nucleic Acids Res.* **22**(22), 4673–4680 (1994). 7984417 10.1093/nar/22.22.4673 PMC308517

[42] Letunic, I. & Bork, P. Interactive tree of life (iTOL) v6: Recent updates to the phylogenetic tree display and annotation tool. *Nucleic Acids Res.* **52**(W1), W78–W82 (2024). 38613393 10.1093/nar/gkae268 PMC11223838

[43] Mackrill, J., Jennings, P. & Cain, R. Exploring positive hospital ward soundscape interventions. *Appl. Ergon.* **45**, 1454–1460 (2014). 24768090 10.1016/j.apergo.2014.04.005

[44] Willis, C., Morley, R., Westbury, J., Greenwood, M. & Pallett, A. Evaluation of ATP bioluminescence swabbing as a monitoring and training tool for effective hospital cleaning. *Br. J. Infect. Control* **8**(5), 17–21 (2007).

[45] Sanna, T. et al. ATP bioluminescence assay for evaluating cleaning practices in operating theatres: Applicability and limitations. *BMC Infect. Dis.* **18**(1), 583. 10.1186/s12879-018-3505-y (2018). 30453892 10.1186/s12879-018-3505-y PMC6245901

[46] Nante, N. et al. Effectiveness of ATP bioluminescence to assess hospital cleaning: A review. *J. Prev. Med. Hyg.* **85**(7), 1079–1095 (2022). PMC5584088 28900359

[47] Arkel, A. et al. ATP measurement as an objective method to measure environmental contamination in 9 hospitals in the Dutch/Belgian border area. *Antimicrob. Resist. Infect. Control* **9**(1), 77. 10.1186/s13756-020-00730-9 (2020). 32466792 10.1186/s13756-020-00730-9 PMC7254657

[48] Ashokan, A. et al. Environmental dynamics of hospital microbiome upon transfer from a major hospital to a new facility. *J. Infect.* **83**, 637–643 (2021). 34606783 10.1016/j.jinf.2021.09.020

[49] Lax, S. et al. Bacterial colonization and succession in a newly opened hospital. *Sci. Transl. Med.* **9**, eaah6500. 10.1126/scitranslmed.aah6500 (2017). 28539477 10.1126/scitranslmed.aah6500 PMC5706123

[50] Vainio-Kaila, T., Kyyhkynen, A., Viitaniemi, P. & Siitonen, A. Pine heartwood and glass surfaces: Easy method to test the fate of bacterial contamination. *Eur. J. Wood Wood Prod.* **69**, 391–395 (2011).

[51] Vainio-Kaila, T. et al. Effect of extractives and thermal modification on antibacterial properties of Scots pine and Norway spruce. *Int. Wood Prod. J.* **4**, 248–252 (2013).

[52] Laireiter, C. et al. Active anti-microbial effects of larch and pine wood on four bacterial strains. *BioResources* **9**(1), 273–281 (2014).

[53] Smailagic, A. et al. Radical scavenging and antimicrobial properties of polyphenol rich waste wood extracts. *Foods* **9**(3), 319. 10.3390/foods9030319 (2020). 32164204 10.3390/foods9030319 PMC7143368

[54] Postovoitova, A., Myronycheva, O. & Karlsson, O. Assessment of the relationships between mould growth characteristics, surface extractive content, and drying methods of Scots pine wood using multivariate data analysis. *Eur. J. Wood Wood Prod.* **83**, 165. 10.1007/s00107-025-02315-y (2025).

